# Do not stop effective atrial-based antitachycardia pacing: Insights into episode duration and success rate for termination

**DOI:** 10.1016/j.hrcr.2024.08.016

**Published:** 2024-08-16

**Authors:** Satoshi Yanagisawa, Yuki Sato, Atsuya Shimizu, Yuji Narita, Yasuya Inden, Toyoaki Murohara

**Affiliations:** 1 Department of Cardiology, Nagoya University Graduate School of Medicine, Nagoya, Japan; 2 Department of Advanced Cardiovascular Therapeutics, Nagoya University Graduate School of Medicine, Nagoya, Japan; 3 Department of Clinical Engineering, Nagoya University Hospital, Nagoya, Japan; 4 Department of Cardiology, National Center for Geriatrics and Gerontology, Obu, Japan; 5 Department of Cardiac Surgery, Nagoya University Graduate School of Medicine, Nagoya, Japan

**Keywords:** Atrial fibrillation, Atrial-based antitachycardia pacing, Termination, Heart failure, Reactive atrial antitachycardia pacing, Pacemaker

**Key Teaching Points**

• Second-generation atrial-based antitachycardia pacing (reactive ATP [rATP]), which has been updated for individual atrial fibrillation (AF) cycle length soon after AF initiation, represents a therapeutic avenue for mitigating AF burden in patients equipped with cardiac implantable electrical devices.
• We present a unique case featuring an uncommon scenario wherein rATP effectiveness transitioned from on to off operation inversely.
• This case highlights concealed efficacy of rATP in managing numerous short AF episodes, which became evident on cessation of therapy and the subsequent increase in ventricular pacing.

## Introduction

Second-generation atrial-based antitachycardia pacing (rATP) represents a therapeutic avenue for mitigating atrial fibrillation (AF) burden in patients equipped with cardiac implantable electrical devices. When the rATP algorithm detects an AF episode, the device tracks rhythm transitions based on regions consisting of atrial cycle length (CL) and regularity. A maximum of 3 antitachycardia pacing therapies (eg, ramp or burst+) with various numbers of sequences can be delivered to each region with a CL in the 50-millisecond range for regular rhythms and 100- to 150-millisecond range for irregular rhythms. When an AF episode persists, antitachycardia pacing can be applied again following a flexible interval to reset the counters (eg, every 8 hours). rATP not only averts progression to chronic AF, but it also ameliorates prognosis and reduces mortality and heart failure (HF) hospitalizations.^1^^,^^2^ We present a unique case featuring an uncommon scenario wherein rATP effectiveness transitioned from on to off operation inversely. This case highlights the concealed efficacy of rATP in managing numerous short AF episodes, which became evident on cessation of therapy and the subsequent increase in ventricular pacing.

## Case reports

A 74-year-old woman presented to an outpatient clinic with chest discomfort. She had undergone mitral valve plasty and dual-chamber pacemaker implantation (Advisa DR MRI, Medtronic, Minneapolis, MN) for sick sinus syndrome 7 years previously. During implantation, the atrial and ventricular transvenous leads were fixed to the right atrial appendage and right ventricular apex, respectively. As the atrioventricular nodal function in the patient was preserved, with a PR interval of 184 milliseconds on the electrocardiogram, the pacing mode was set with managed ventricular pacing to maintain intrinsic atrioventricular conduction. The lower rate of the pacemaker was adjusted to 60 beats/min. Four years earlier, AF was evident; thus, we introduced rATP equipped with a Medtronic pacemaker system in this patient. Her medications included bisoprolol (2.5 mg) and apixaban (10 mg) daily, which were continued thereafter. Although the interval from atrial pacing to ventricular sensing in the interrogation has gradually increased to 300–400 milliseconds in recent years, the patient could continue the managed ventricular pacing mode with an intrinsic ventricular rhythm using the manufacturer's nominal setting: the ability to observe a maximum atrioventricular interval of 420 milliseconds or mean atrioventricular interval plus 100 milliseconds (<600 milliseconds). On echocardiography, enlarged left atrial diameter and left ventricular end-diastolic and -systolic diameters detected preoperatively were significantly improved at 1-month postoperative echocardiography (left atrial diameter decreased from 40.8 mm to 35.3 mm, while left ventricular end-diastolic and -systolic diameters decreased from 56.6 mm and 32.2 mm to 47.9 mm and 30.6 mm). Thereafter, no remarkable changes in these parameters were observed on 2-year follow-up echocardiography (left atrial diameter, 36.9 mm; left ventricular end-diastolic and -systolic diameters, 48.7 mm and 31.9 mm).

**Figure 1:**
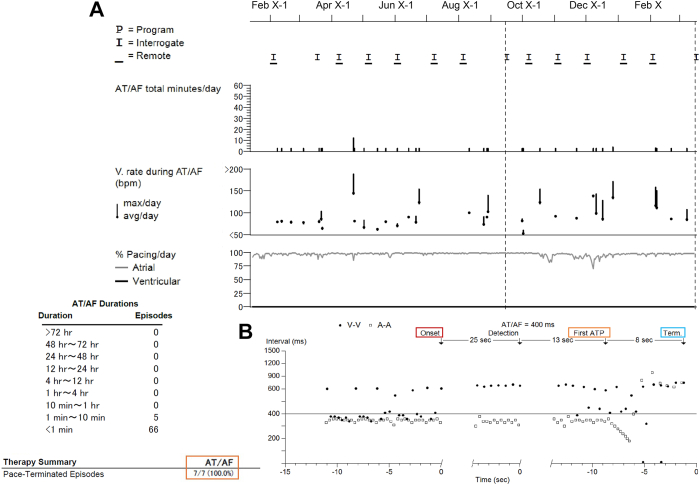
Pacemaker interrogation for atrial fibrillation (AF) burden before and after the visit. **A:** During the 6 months before the visit, AF occurrences were infrequent, with short durations of a few minutes, and the success rate of atrial-based antitachycardia pacing (rATP) termination was 100%. **B:** A representative episode of AF successfully terminated by rATP using the first sequence is depicted. The total duration of the AF episodes was within 1 minute from onset to termination. AT/AF = atrial tachycardia/atrial fibrillation.

**Figure 2:**
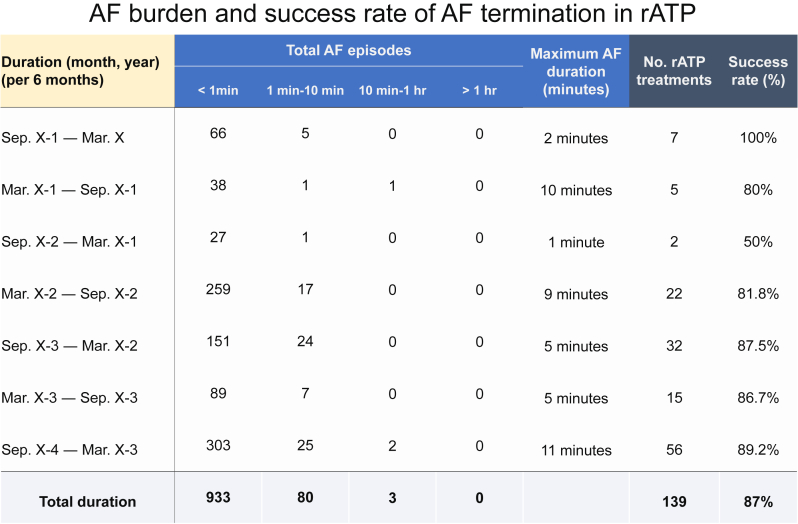
Atrial fibrillation (AF) episodes and success rate of AF termination in the atrial-based antitachycardia pacing (rATP) before 4 years. Interrogation records from the pacemaker over the past 4 years illustrate that the majority of AF episodes lasted within 10 minutes, with a notable mean success rate of 87% for rATP termination during this period.

During the present examination and device interrogation, an interval of atrial pacing to ventricular sensing was prolonged by >400 milliseconds in the AAIR mode, which was a possible cause of her symptoms. Thus, we changed the pacing mode to DDDR and discontinued rATP based on the lower AF burden in the recent device record. Indeed, recent AF occurrences were infrequent, with short durations of only a few minutes, and the success rate of rATP termination was 100% (Figure 1). Interrogation records from the pacemaker over the past 4 years illustrated that most AF episodes lasted within 10 minutes, with a notable mean success rate of 87% for rATP termination (Figure 2). It is also noted that the patient had no history of HF hospitalization post pacemaker implantation, and recent echocardiography indicated a preserved left ventricular ejection fraction of 63%.

**Figure 3:**
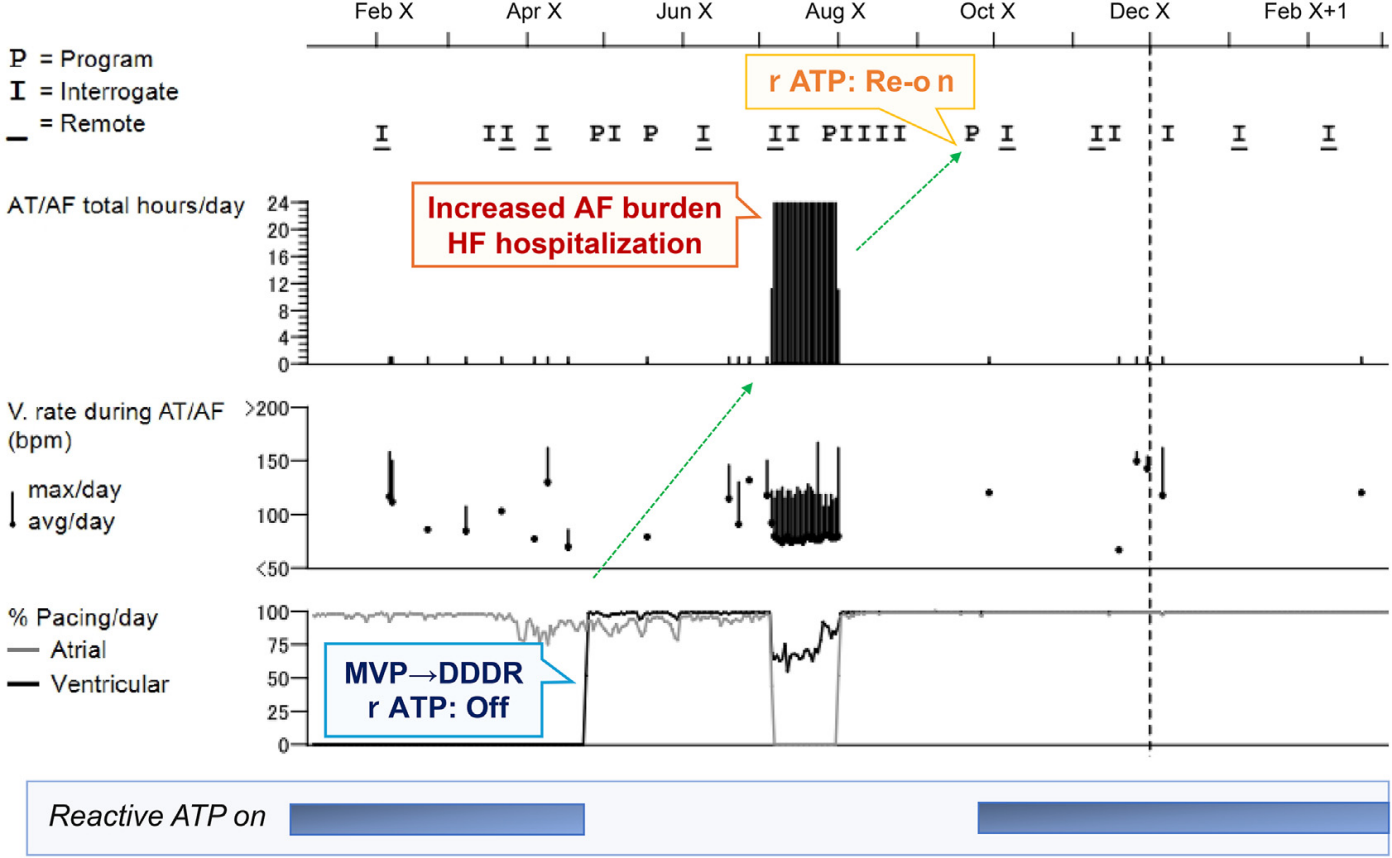
Timeline of events and treatments following pacing mode change and discontinuation of atrial-based antitachycardia pacing (rATP). AF = atrial fibrillation; AT = atrial tachycardia; HF = heart failure; MVP = managed ventricular pacing.

The patient showed a slight improvement in her chest discomfort after the 1-week visit; therefore, we continued the DDDR pacing mode. However, 3 months after the cessation of rATP and transitioning to the DDDR pacing mode, the patient was hospitalized for HF alongside persistent AF lasting 3 weeks. HF management and administration of sodium-glucose transport protein 2 inhibitors were initiated. We also attempted to terminate the AF with a CL of 200 milliseconds by manual antitachycardia pacing from the atrial lead at the time of admission; however, the AF did not terminate. Following administration of oral pilsicainide for 1 week, we attempted manual antitachycardia pacing again for AF with relatively regular tachycardia and longer CL (220–230 milliseconds), and the AF was terminated successfully. Subsequently, rATP was reactivated, resulting in AF suppression for 6 months after discharge (Figure 3). Although this patient was recommended to receive catheter ablation for the symptomatic AF, we have not obtained agreement from her at this point.

## Discussion

Conventionally, the efficacy of rATP is confirmed by initiating the therapy against a frequent AF burden and subsequently observing a decline in AF occurrences.^2^ However, we present herein a unique case featuring an uncommon scenario in which rATP effectiveness transitioned from on to off operation inversely.

In this case, the development of persistent AF may have been precipitated by the cessation of rATP therapy, and it is also plausible that the increased ventricular pacing rate with DDDR mode might influence AF development, despite reported annual AF development rates in cardiac implantable electronic devices not exceeding 6.6%.^3^ Because of the majority of AF episodes appearing short over the long term and the infrequent occurrence of AF in the last 6 months, a decision was made to discontinue rATP therapy to mitigate the additional risks of battery depletion and AF acceleration.^4^ However, the remarkably high success rate of rATP played a pivotal role in suppressing AF progression during early stages, effectively concealing all episodes within brief AF durations. This case highlights the concealed and invaluable role of rATP in curtailing AF progression, particularly during numerous episodes of brief AF.

Once AF persists, the success rate of conversion to sinus rhythm decreases with AF persistence duration. AF may induce electrical and structural remodeling of the atrium, leading to its maintenance and stability. This feature is well-explained by a phenomenon called “AF begets AF.”^5^ Perhaps the failure of the manual antitachycardia pacing at the time of admission, in this case, may have been caused by persistent AF for several weeks with the progression of the atrial remodeling. In this regard, repetitive attempts of the second-generation rATP, which has been updated for individual AF-CL soon after AF initiation, can make it possible to prevent persistent AF at an early stage, and continuous rATP might have suppressed AF progression. The result was also shown in the MINERVA study, a prospective large clinical trial reporting the efficacy of managed ventricular pacing plus rATP in preventing conversion to persistent AF.^1^ Furthermore, rATP was more effective with an increased success rate for terminating AF with slow CL and regular tachycardia,^6^^,^^7^ and the beneficial result was also demonstrated in a specific population undergoing catheter ablation therapy in our previous study.^2^ In this case, additional administration of oral pilsicainide may prolong the AF-CL and change the AF rhythm more regularly, which may result in the successful termination of AF following manual antitachycardia pacing. Nonetheless, continuous rATP might have prevented HF hospitalization, thereby avoiding additional costs of hospitalizations and emergency visits, which were reported to be reduced with the introduction of rATP,^8^ although this case did not adequately disentangle the direct causal effect of rATP in preventing persistent AF from the temporal relationship between the change in setting and the transition to persistent AF. However, it is worth noting that such persistent AF associated with HF hospitalization never occurred for more than 4 years before admission, and all AF episodes were suppressed within 1 hour in the previous records, suggesting a possible hidden effect of rATP on the outcomes in this case.

Alternatively, this case had a good indication of catheter ablation for the symptomatic AF causing HF hospitalization, although we did not yet perform the procedure, possibly because of her stable clinical course without relevant recurrence after retreatment of rATP and asymptomatic AF episodes over the past 4 years. Although catheter ablation remarkably reduced AF burden and frequency, subclinical asymptomatic recurrence was often noted by the continuous device monitoring.^9^ The rATP may have an additional role in suppressing the remaining AF after the ablation, and combination therapy of prompt catheter ablation and rATP may further reduce AF burden with better clinical outcomes.^2^ Despite approximately 10 years since the multicenter randomized MINERVA trial,^1^ rATP use and efficacy might be underestimated in some clinical situations. Our case report highlights the potential ability of rATP to reduce AF burden and progression to persistent AF, as mentioned in the latest updated guidelines.^10^

## Conclusion

This unique scenario underscores the efficacy of rATP in managing numerous short AF episodes, which became evident on cessation of therapy and a subsequent increase in ventricular pacing. The results of the device interrogation are key to understanding the substantial efficacy of rATP results that should not be discontinued. We delved into a potentially pivotal factor necessitating attention, elucidating its role in fostering rATP efficacy in preventing AF persistence, thereby shedding light on the substantial efforts of rATP behind the images of the interrogation results (Supplemental Figure 1).

## Disclosures

Satoshi Yanagisawa is affiliated with a department sponsored by Medtronic, Japan; the rest of the authors have no conflicts of interest.
