# Supplementary material for: Do not stop effective atrial-based antitachycardia pacing: Insights into episode duration and success rate for termination
Source: HeartRhythm Case Rep. 2024 Aug 16;10(12):867–71. doi: 10.1016/j.hrcr.2024.08.016 (PMC11781891; doi:10.1016/j.hrcr.2024.08.016)
Supplement: Supplemental Figure 1 [file mmc1.docx]

**Supplemental appendix**

Yanagisawa *et al.*: Do not stop effective atrial-based antitachycardia pacing: Insights into episode duration and success rate for termination

**Table of contents**

**Page 2:** *Supplemental Figure 1.* Schematic representation of treatment progression and AF burden before and after discontinuation of rATP therapy

**Supplemental Figure 1**


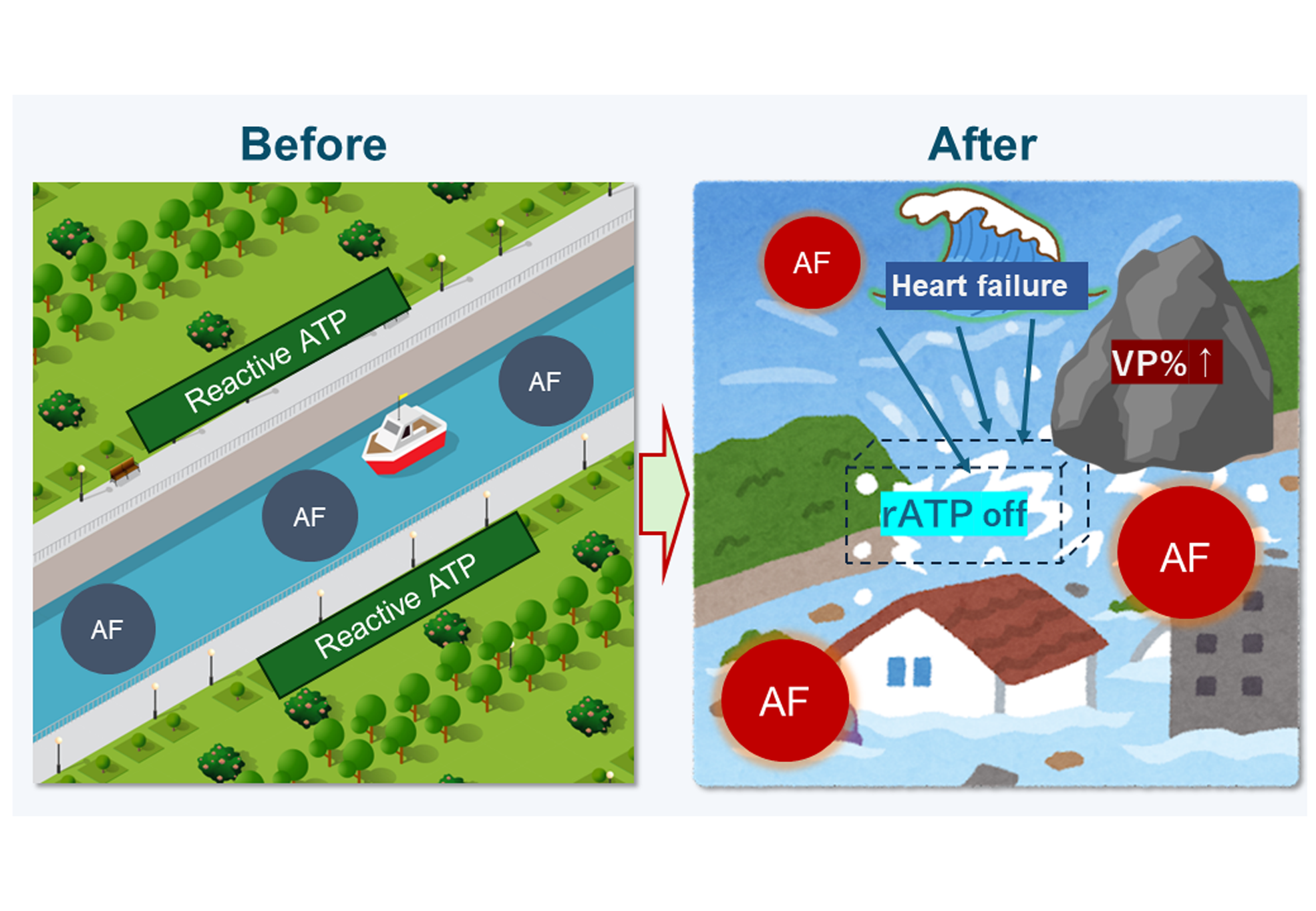


**Supplemental Figure 1. Schematic representation of treatment progression and AF burden before and after discontinuation of rATP therapy**

The development of persistent AF, in this case, may have been precipitated by the cessation of rATP therapy and subsequent increase in ventricular pacing rate, with HF exacerbation perpetuating the AF rhythm thereafter. This unique scenario underscores the efficacy of rATP in managing numerous short AF episodes, which became evident upon the cessation of therapy and the subsequent increase in ventricular pacing. The continuation of effective rATP therapy would have maintained a high success rate, albeit masked by the short duration of AF episodes.

AF, atrial fibrillation; HF, heart failure; rATP, atrial-based anti-tachycardia pacing.
